# BODY IMAGE DURING PUBERTY: WHAT HAPPENS TO HOW KIDS FEEL ABOUT THEIR BODIES?

**DOI:** 10.3389/frym.2024.1178387

**Published:** 2024-03-08

**Authors:** Savannah R. Roberts, Sasha Gorrell, Daniel Le Grange

**Affiliations:** 1 Department of Psychology, University of Pittsburgh, Pittsburgh, PA, United States; 2 Department of Psychiatry and Behavioral Sciences, University of California, San Francisco, San Francisco, CA, United States; 3 Department of Psychiatry and Behavioral Neuroscience, The University of Chicago, Chicago, IL, United States

## Abstract

Adolescence, the period between childhood and adulthood, is a time when significant changes happen to the human body. These changes, called puberty, signify the time during which humans develop into adults. During puberty, changes in weight, height, and muscle size leave many adolescents feeling unhappy with their appearance. We describe how these changes affect body image, which is the way that adolescents feel about their bodies. First, we discuss how a negative body image can be a risk factor for eating disorders and depression. Second, we highlight the body positivity movement, which encourages people to love their bodies no matter what they look like. Lastly, we describe body neutrality, an attitude that celebrates bodies for what they allow us to do, rather than for their appearance. Together, these body image concepts can help people to understand how physical changes that accompany puberty might affect mental health.

## WHAT IS BODY IMAGE?

**Body image** is a term used to refer to how someone feels about their body and the way it looks. Body image can have a large impact on happiness. During **adolescence**, the period between childhood and adulthood that spans roughly ages 10–19, **puberty** can lead to big changes in the way the body looks. Puberty involves the series of transformations that occur as someone becomes an adult, including weight gain and growth spurts. It is normal to wish that you could change something about your body while these changes are happening. However, if a person really dislikes the way they look, they might be tempted to go to extremes (such as excessive dieting or exercise) to try to change it. That can be dangerous! Therefore, it is important to understand your own body image, so that you can learn to appreciate the physical changes that happen during puberty.

## PHYSICAL CHANGES DURING PUBERTY

Puberty leads to two main physical changes: height and weight gain; and growth of **secondary sex characteristics** [1]. Both of these changes can impact young people’s body image.

During puberty, people experience changes in their body proportions. Girls grow about 3.5 inches per year, while boys grow about 4 inches per year [1]. Girls typically experience this growth spurt 2 years earlier than boys, who “catch up” around age 14 [1]. The head, hands, and feet are among the first body parts to grow, followed by the arms, legs, chest, and shoulders. On average, people also gain half of their adult body weight during adolescence [2]. In boys, weight gain is about 75% muscle and 25% body fat, while in girls, weight gain is about 55% body fat and 45% muscle [2].

Puberty also includes the development of secondary sex characteristics, or parts of the body that are not directly required for reproduction [1]. While every adolescent experiences these changes, the age of onset and pace differ from person to person. Girls develop hips and breasts, whereas boys develop a bump on the throat called the Adam’s apple. Both girls and boys start growing thicker, darker hair between their legs and in their armpits. Boys also grow hair on their faces and chests, and their voices deepen. During puberty, glands in the skin produce more oil, causing acne. Although all these changes are normal, they can have a big impact on an adolescent’s body image.

## TYPES OF BODY IMAGE

**Figure 1:**
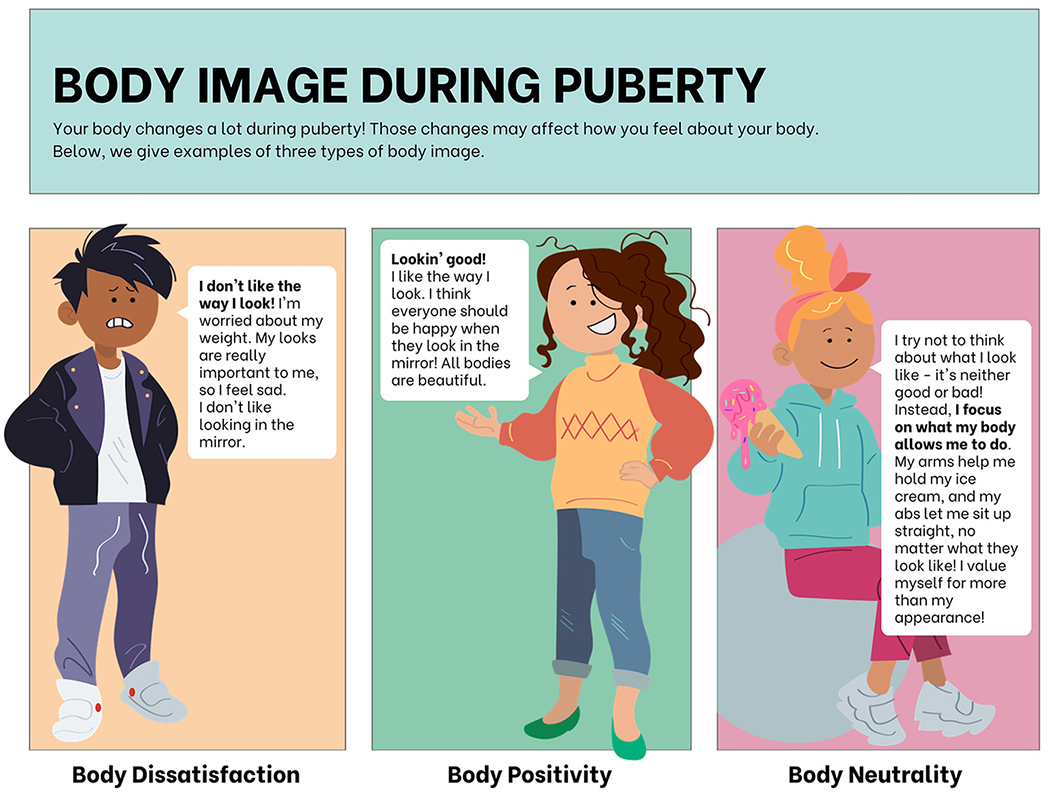
Types of body image that adolescents may experience during puberty.

During puberty, a person’s body image may change. Below, we compare three types of feelings that someone could have about the ways in which their body is developing (Figure 1).

### Body Dissatisfaction

With all the ways the body changes, adolescents may struggle to accept their new appearance. When someone does not like something about their body or appearance, it is called a negative body image, or **body dissatisfaction**. In girls, body dissatisfaction often shows up as unhappiness with changes to their stomachs and waists. Many girls worry about the weight they gain during puberty, even though it is a normal part of getting older [3]. Boys may also feel body dissatisfaction. For example, some boys wish they could gain more muscle and do not like feeling “scrawny”. Boys and girls may start getting more attention from peers and might feel self-conscious about their bodies. Young people who are teased about their weight are especially likely to feel body dissatisfaction [4]. While body dissatisfaction is a normal feeling, it can lead adolescents to adopt unhealthy diet and exercise behaviors in an attempt to change their appearance.

### Body Positivity

When someone likes something about the way their body looks, it is called a positive body image, or **body positivity**. There is more to body positivity than what you may have seen on social media [5]! Body positivity encourages people to value their bodies no matter their weight, size, or looks. In other words, your body is beautiful just the way it is!

Compared to girls, boys generally have more body positivity during puberty. During puberty, boys can build muscle easier than ever before, and they are often happy to grow a few inches taller [1]. Boys who go through puberty earlier than their friends are especially likely to feel body positivity. Girls can also feel good about the changes happening to their bodies. For example, some girls might like the curves and strength they gain. For boys and girls, changes that happen during puberty can help with athletics and sports, which may increase their popularity.

### Body Neutrality

So far, we have talked about two kinds of body image: body negativity and body positivity. These types of body image refer to the way someone feels about their *looks*. However, there is much more to the body than its appearance! **Body neutrality** encourages people to focus on what their bodies allow them to do, instead of what their bodies look like [5]. Body neutrality also encourages people to think of their bodies with a neutral point of view, not with negativity or positivity. For example, if a person is unhappy with the way their stomach looks, they could try body neutrality, and appreciate how their stomach holds in their organs and keeps them alive—no matter what it looks like! Now you try—can you think of a part of your body that you dislike or feel insecure about? What would it be like to practice body neutrality? For people who do not like their bodies, body neutrality can feel more realistic and achievable than body positivity. Even if you are not quite sure how you feel about your body as a whole, see if you can honor and respect a part of your body for what it does for you, without trying to feel positive or negative about the way it looks.

## WHY DOES BODY IMAGE MATTER?

How adolescents think and feel about their bodies has a large impact on their mental health. As we mentioned before, many adolescents become unhappy or feel out of control when their bodies start changing, and body dissatisfaction is usually at its highest during puberty. Yet, when adolescents feel good about their bodies, they often have better self-esteem and overall well-being. Therefore, it is important to understand the negative things that might happen when young people struggle with body dissatisfaction, so that we can help them by learning ways to promote a healthy and positive body image.

Because body image is related to mood, young people are at a higher risk for **eating disorders** and depression during puberty [1]. Young people who experience body dissatisfaction have the highest risk for eating disorders. Eating disorders are mental illnesses in which people try to change their weight or shape in unhealthy ways. They can be very harmful to a person’s health, so it is important to stop them in their tracks as soon as they start! Poor body image can lead someone to try to limit the amount or the types of foods they eat. Sometimes people may feel guilty about the number of calories they have eaten, which can lead to a desire to get rid of those calories with excessive exercise or other unhealthy behaviors. Although some of these behavior changes may start slowly, body dissatisfaction can quickly lead to full-blown eating disorders.

Even when a person does not develop an eating disorder, they still may feel like they will never be happy with how they look, and their self-esteem can be impacted. They might become more self-critical and feel hopeless that things will never change. It is easy to see how this could lead someone to be in a bad mood most of the time! Therefore, combatting body dissatisfaction is critical during adolescence. Promoting body positivity and body neutrality can improve young people’s mental health and emotional well-being. Fostering a healthy body image empowers adolescents to embrace the changes happening to their bodies with confidence, improving mood and supporting a more positive mindset. If you or someone you know is struggling with negative feelings about their body, eating, or low mood, it is important to consult with a trusted adult who can help find helpful support and resources. To start, we recommend checking the National Eating Disorders Association’s website.

## UNDERSTANDING BODY IMAGE CHANGES DURING PUBERTY

Body image is a key concept when it comes to understanding and navigating adolescence. Puberty is a time of huge physical changes, which can lead young people to evaluate themselves based on how they look. How people think about their bodies is directly related to their mental health. Body dissatisfaction may put people at greater risk of struggling with eating and exercise or with depression. Sometimes, having a more positive body image allows people to love their bodies despite all the changes! Alternatively, a more neutral focus on the function of the body can help adolescents appreciate how their bodies allow them to do all of the activities that make life so fun. Together, no matter how adolescents feel about their bodies, an understanding of the types of body image and how they arise can provide a helpful framework for understanding some of the challenges of puberty.

## Abbreviations

BODY IMAGE: How someone feels about their body or their appearance.
ADOLESCENCE: The period between childhood and adulthood that spans roughly ages 10–19.
PUBERTY: A biological process that normally starts in adolescence, when hormonal changes allow a person to become sexually mature.
SECONDARY SEX CHARACTERISTICS: Parts of the body that develop during puberty that are not directly required for reproduction, such as breasts, hips, and facial hair.
BODY DISSATISFACTION: When someone holds negative thoughts about their body or appearance.
BODY POSITIVITY: When someone holds a positive view of their body, regardless of size, shape, or other physical characteristics.
BODY NEUTRALITY: When someone appreciates what their body allows them to do, no matter what it looks like.
EATING DISORDERS: Serious mental health conditions where people have unhealthy thoughts, feelings, and behaviors about food and their bodies.
